# Association of marital relationship with quality of life among older adults with mild cognitive impairment and mild dementia

**DOI:** 10.1111/ggi.14868

**Published:** 2024-04-23

**Authors:** Chisato Fujisawa, Hirotaka Nakashima, Hitoshi Komiya, Kazuhisa Watanabe, Yosuke Yamada, Tomihiko Tajima, Hiroyuki Umegaki

**Affiliations:** ^1^ Department of Community Healthcare and Geriatrics, Graduate School of Medicine Nagoya University Nagoya Japan; ^2^ Institute of Innovation for Future Society Nagoya University Nagoya Japan

## Abstract

The marital relationship is associated with the quality of life among those with cognitive impairment, but sarcopenia status seems to play an important role in the association.

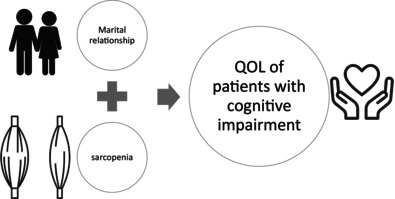

Dear Editor,

Evaluating quality of life (QOL) among older adults with cognitive impairment (CI) has become increasingly important in considering where they should spend their remaining years. Given that older adults with CI spend more time at home with their spouses^1^ owing to retirement and their children's independence, we hypothesized that the marital relationship would play an important role in QOL. In the current study, we examined the association between marital relationship and QOL among older adults with CI.

This study included 35 patients aged 65 years or older who attended outpatient gerontology centers or the Geriatrics Department of Nagoya University Hospital for forgetfulness from May 2019 to June 2023. The inclusion criteria were a Mini‐Mental State Examination (MMSE) score of 20 or over and visiting the clinic with their spouse, in order to obtain more accurate information regarding the patient's cognitive status. QOL was assessed using both QOL for Patients Receiving Home‐Based Medical Care (QOL‐HC)^2^ and the EuroQol Five‐Dimension Scale (EQ‐5D), which has been validated in Japanese patients.^3^ The marital relationship was evaluated by the amount of conversation per day between the couple (≥1 h or <1 h); how often the couple traveled, went shopping, ate out, and did hobbies together (hardly ever, occasionally, and often); and the total score for marital satisfaction on a scale from 0 (very dissatisfied) to 10 (very satisfied).^4^ Marital satisfaction was rated by patients. LGBT couples were not included.

A multiple linear regression model was used to examine the association between these marital relationship variables and QOL variables, with adjustment for age, sex, and sarcopenia status. Multicollinearity was assessed using correlation coefficients and variance inflation factors. Sarcopenia was diagnosed according to the Asian Working Group for Sarcopenia 2019 criteria.^5^ Statistical analysis was performed using SPSS (version 29.0). A *P*‐value of less than 0.05 was considered statistically significant. This study was approved by the Ethical Committee of Nagoya University.

Patient characteristics are shown in Table S1. The mean patient age was 78.8 years, and 19 (54.3%) patients were women. The mean MMSE score was 24.3. Three patients (8.6%) had sarcopenia, and nine (25.7%) had severe sarcopenia. As shown in Table 1, patients who talked with their spouses for more than 1 h per day, those who traveled together at least occasionally, and those who had higher marital satisfaction had a higher QOL‐HC score, although no models were statistically significant. When sarcopenia status was added as a covariate, the association between marital relationship and QOL‐HC score was no longer significant. On the other hand, patients who traveled together at least occasionally had a higher EQ‐5D score, but the models were not significant, even after adjusting for age, sex, and sarcopenia status. Sarcopenia status was not significantly associated with any of the marital relationship variables.

**Table 1:** Correlation between QOL and marital relationship

Couple activities	Covariates: age and sex				Covariates: age, sex, and sarcopenia			
	Adjusted β	*P*	*R* ^2^	*F*	Adjusted β	*P*	*R* ^2^	*F*
Subjective QOL‐HC score								
At least 1 h of conversation per day	0.4	0.02	0.1	2.4	0.3	0.2	0.3	3.5*
Traveling at least occasionally	0.4	0.02	0.1	2.6	0.3	0.2	0.3	3.8*
Going shopping at least occasionally	0.02	0.9	−0.1	0.3	0.1	0.6	0.3	3.1*
Eating out at least occasionally	0.2	0.4	−0.04	0.6	0.3	0.1	0.3	4.2*
Doing hobbies together at least occasionally	0.1	0.6	−0.1	0.5	−0.1	0.5	0.3	3.3*
Marital satisfaction of patients	0.4	0.04	0.1	2.0	0.1	0.6	0.2	2.9*
EQ‐5D‐5L								
At least 1 h of conversation per day	0.1	0.7	−0.1	0.1	0.01	0.95	−0.2	0.1
Traveling at least occasionally	0.4	0.03	−0.04	0.6	0.07	0.04	0.1	0.3
Going shopping at least occasionally	0.2	0.3	−0.04	0.6	0.3	0.1	−0.03	0.8
Eating out at least occasionally	−0.04	0.9	−0.08	0.2	0.04	0.9	−0.1	0.2
Doing hobbies together at least occasionally	0.2	0.3	−0.1	0.5	0.2	0.4	−0.1	0.4
Marital satisfaction of patients	0.2	0.2	−0.02	0.8	0.02	0.9	−0.1	0.3

*Note*: *R* ^2^ and *F* were for each multiple regression model equation.

* Significance level *P* ≤ 0.05 for each multiple regression model.

These results suggest that the marital relationship is correlated with QOL among older adults with CI when adjusting for only age and sex, but that sarcopenia status is significantly involved in the correlation.

QOL‐HC was developed because of the difficulty of QOL assessment among people who are physically frail, and it focuses on factors other than physical function, such as perceived social support and relationships with surroundings.^2^ On the other hand, EQ‐5D measures functional capacity, capacity for social activities, and daily health status. This could explain the results that the marital relationship assessed in terms of conversation time was more strongly correlated with QOL‐HC than with EQ‐5D score when adjusting for age and sex, and that patients who could travel with their spouse at least occasionally had a better EQ‐5D score.

The small number of participants in this study may have influenced the results. Future longitudinal studies with a larger number of couples are needed in order to more broadly reflect the range of behavioral and psychological symptoms of dementia and other psychological issues. Nevertheless, this study is the first to consider the association between marital relationship and QOL among older adults with mild CI. Our results support the idea that physical frailty, such as sarcopenia, has an impact on the association between marital relationship and QOL.

This study suggests that the marital relationship is involved in QOL among those with CI, but sarcopenia status seems to play an important role in the association between marital relationship and QOL. Further studies on the correlation between marital relationship and QOL are needed.

## Disclosure statement

The authors declare no conflict of interest.

## Supporting information


**Table S1.** Patients characteristics (*N* = 35).

## Data Availability

The data that support the findings of this study are available on request from the corresponding author. The data are not publicly available due to privacy or ethical restrictions.
