# Supplementary material for: Association of marital relationship with quality of life among older adults with mild cognitive impairment and mild dementia
Source: Geriatr Gerontol Int. 2024 Apr 23;24(6):646–7. doi: 10.1111/ggi.14868 (PMC11503544; doi:10.1111/ggi.14868)
Supplement: Supplementary file 1 — Table S1. Patients characteristics (N = 35). [file GGI-24-646-s001.docx]

Supplemental Table 1. Patients characteristics (N=35)

|  | Median (95%) or n (%) |
| --- | --- |
| Age, years | 78.8 (6.8) |
| Women | 19 (54.3) |
| Education, years | 12.5 (2.9) |
| Basic activities of daily living | 99.4 (2.0) |
| Sarcopenia status |  |
| Sarcopenia | 3 (8.6) |
| Severe sarcopenia | 9 (25.7) |
| Family income |  |
| ≤1 million yen per year | 1 (2.9) |
| 1-3 million year per year | 17 (48.6) |
| 3-5 million year per year | 12 (34.3) |
| ≥5 million per year | 1 (2.9) |
| No answer | 4 (11.4) |
| Mini-Mental State Examination | 24.3 (2.0) |
| Diagnosis |  |
| Mild cognitive impairment | 21 (60.0) |
| Alzheimer disease | 11 (31.4) |
| Lewy body dementia | 1 (2.9) |
| Other | 2 (5.7) |
| Nursing care certification |  |
| Using care service with certification | 5 (14.3) |
| Not using care service with certification | 30 (85.7) |
| Currently driving | 8 (22.9) |
| Nursing care level |  |
| 0 | 30 (85.7) |
| 1 | 1 (2.9) |
| 2 | 1 (2.9) |
| 3 | 2 (5.7) |
| 4 | 1 (2.9) |
| Charlson Comorbidity Index |  |
| 0 | 9 (25.7) |
| 1 | 12 (34.3) |
| 2 | 5 (14.3) |
| 3 | 6 (17.1) |
| 4 | 1 (2.9) |
| 5 | 2 (5.7) |
| Relationship with spouse |  |
| At least 1 h of conversation per day | 18 (51.4) |
| Travel at least occasionally | 20 (60.0) |
| Go to shopping at least occasionally | 27 (77.1) |
| Eat out at least occasionally | 23 (65.7) |
| Doing hobbies together at least occasionally | 16 (51.4) |
| Marital satisfaction | 8.2 (1.7) |
| QOL-HC score | 6.6 (1.0) |
| EQ-5L score | 0.9 (0.1) |
